# Diabetes and Sarcopenia: Metabolomic Signature of Pathogenic Pathways and Targeted Therapies

**DOI:** 10.3390/ijms26157574

**Published:** 2025-08-05

**Authors:** Anamaria Andreea Danciu, Cornelia Bala, Georgeta Inceu, Camelia Larisa Vonica, Adriana Rusu, Gabriela Roman, Dana Mihaela Ciobanu

**Affiliations:** 1 Ph.D. School, “Iuliu Haṭieganu” University of Medicine and Pharmacy, 400006 Cluj-Napoca, Romania; gorzoanamaria@yahoo.com; 2 Diabetes Center, Emergency Clinical County Hospital Cluj, 400006 Cluj-Napoca, Romania; inceu.victoria@umfcluj.ro (G.I.); adriana.rusu@umfcluj.ro (A.R.); groman@umfcluj.ro (G.R.); dana.ciobanu@umfcluj.ro (D.M.C.); 3 Department of Diabetes and Nutrition Diseases, “Iuliu Haṭieganu” University of Medicine and Pharmacy, 400006 Cluj-Napoca, Romania; camelia.sulea@umfcluj.ro

**Keywords:** metabolomics, diabetes mellitus, sarcopenia, therapeutic approach, narrative review

## Abstract

Diabetes mellites (DM) is a chronic disease with increasing prevalence worldwide and multiple health implications. Among them, sarcopenia is a metabolic disorder characterized by loss of muscle mass and function. The two age-related diseases, DM and sarcopenia, share underlying pathophysiological pathways. This narrative literature review aims to provide an overview of the existing evidence on metabolomic studies evaluating DM associated with sarcopenia. Advancements in targeted and untargeted metabolomics techniques could provide better insight into the pathogenesis of sarcopenia in DM and describe their entangled and fluctuating interrelationship. Recent evidence showed that sarcopenia in DM induced significant changes in protein, lipid, carbohydrate, and in energy metabolisms in humans, animal models of DM, and cell cultures. Newer metabolites were reported, known metabolites were also found significantly modified, while few amino acids and lipids displayed a dual behavior. In addition, several therapeutic approaches proved to be promising interventions for slowing the progression of sarcopenia in DM, including physical activity, newer antihyperglycemic classes, D-pinitol, and genetic USP21 ablation, although none of them were yet validated for clinical use. Conversely, ceramides had a negative impact. Further research is needed to confirm the utility of these findings and to provide potential metabolomic biomarkers that might be relevant for the pathogenesis and treatment of sarcopenia in DM.

## 1. Introduction

Diabetes mellitus (DM) is a chronic disease with increasing prevalence worldwide as it has been announced by the newest 11th edition of the International Diabetes Federation Diabetes Atlas in 2025. Being one of the fastest-growing non-communicable health challenges of this century, DM is estimated to affect 589 million adults aged 20 to 79 years, of whom an overwhelming 43% are undiagnosed. As DM prevalence increases with age, the percentage of older people above 65 years affected now estimated at 23.7% is expected to increase, particularly in aging societies [1]. Disability-adjusted life years (DALYs) attributable to DM presented a significantly increasing trend globally from 28.6 million in 1990 to 70.9 million in 2019. The numbers reported in the last three decades are expected to increase in the future [2].

Sarcopenia is an under-recognized metabolic disorder characterized by loss of muscle mass and function, which impacts aging populations globally and has multiple identifiable risk factors [3,4]. Among them, aging and DM play significant roles. Muscle metabolism alteration and insufficiency occurring with advancing age is associated with the switch of subcutaneous fat with an abnormal localization of fat in the skeletal muscles. DM induces alteration of the lipid metabolism in the skeletal muscle by increasing the fat accumulation, also called myosteatosis. The lipotoxic environment created by myosteatosis reduces the muscle mass and strength leading to typical features of sarcopenia [5]. Also, muscle alteration is accelerated by oxidative stress, accumulation of advanced glycation end-products, and chronic inflammation specific to DM. In turn, impaired muscle health, as a result of sarcopenia, worsens glycemic control leading to the development and progression of DM. The two aged-related diseases, DM and sarcopenia, share underlying pathophysiological pathways [6,7,8], and their mutual interrelationship generated the term DM associated with sarcopenia. A recent study involving participants from the National Health and Nutrition Examination Survey reported that the prevalence of sarcopenia was significantly higher in the presence of type 2 DM compared with subjects without DM above the age of 50 years—27.9% versus 15.7% [9]. On the other hand, sarcopenia is associated with a two-fold increase in the chance of having DM [10]. Sarcopenia is one of the conditions encompassed under the umbrella term musculoskeletal disorders, also known for increasing incidence globally and reported to have had a DALYs of 42 million in 1990 and a DALYs of 68.5 million in 2021, resulting in an increase of over 60% in the last three decades [11].

Targeted and untargeted metabolomic studies performed in humans with DM, as well as in animal models of DM and cell cultures, revealed significant changes in carbohydrate, lipid, protein, and energy metabolisms [12,13]. Metabolic alterations were also reported in sarcopenia [14,15,16,17]. For instance, fatty acids and sphingolipid metabolism were associated with development and progression of both DM [18,19] and sarcopenia [20,21]. However, a clear description of sarcopenia in DM using a recent metabolomic approach is still lacking.

**Figure 1:**
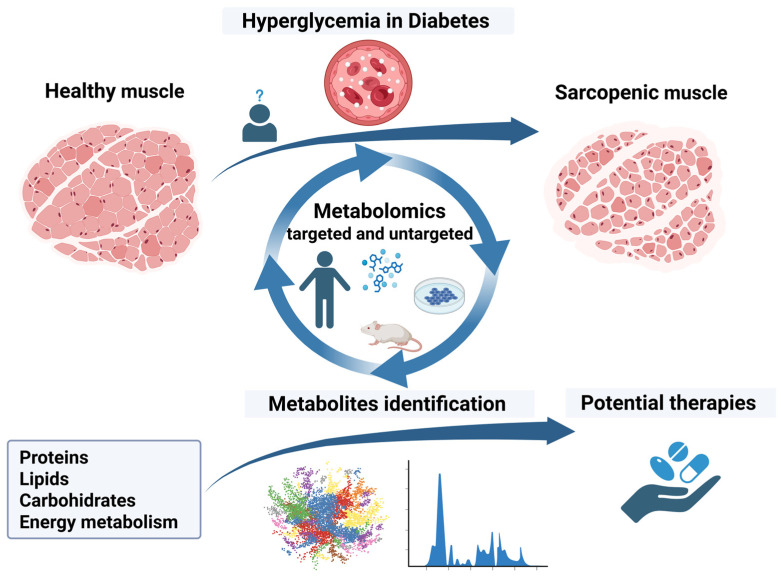
Diabetes and sarcopenia in studies using metabolomic analysis. Created in BioRender. Ciobanu, D. (2025) https://BioRender.com/3xi0f1n (accessed on 15 July 2025).

This narrative review will explore the current evidence from studies using complex and different metabolomic techniques utilized for identifying and describing biomarkers related to sarcopenia in DM. Furthermore, the potential role of several therapeutic approaches to alleviate sarcopenia in DM will be presented. Figure 1 graphically presents the aims of the narrative review.

## 2. Key Findings from Metabolomic Studies in Sarcopenia Associated with Diabetes

Advancements in metabolomic analytical techniques have made possible the simultaneous identification and quantification of multiple biomarkers. Metabolic perturbations associated with hyperglycemia were widely explored to find potential biomarkers associated with DM. We searched for evidence investigating the association of DM with sarcopenia using targeted and untargeted metabolomic techniques in both humans and animal models of DM, as well as cell cultures.

### 2.1. Amino Acids, Peptides and Proteins

Amino acids, peptides, and proteins were investigated as potential biomarkers for sarcopenia and different amino acids profiles have been associated with different sarcopenia phenotypes. A cross-sectional study by Tan et al. provides an extensive non-targeted metabolomic analysis in patients with type 2 DM with sarcopenia (*n* = 10) and type 2 DM without sarcopenia (*n* = 10). Using ultra-high-performance liquid chromatography electrospray-ionization tandem mass spectroscopy (UHPLC-ESI-MS/MS), 632 serum metabolites were identified, but only 82 were significantly altered between the groups with type 2 DM with and without sarcopenia. Patients with type 2 DM and sarcopenia presented significantly increased levels of 5′-methylthioadenosine (5′-MTA), asymmetric dimethylarginine, N,N-dimethylarginine, and glutamine. It is notable that isoxanthohumol was considerably decreased [22]. 5′-MTA is an intermediate metabolite of the methionine cycle and polyamine synthetase, and it is known that high levels of methionine can cause skeletal muscle injury and dysfunction and can reduce skeletal muscle development and growth [23]. Asymmetric dimethylarginine, an endogenous inhibitor of nitric oxide synthesis, was remarkably increased in the group of patients with type 2 DM with sarcopenia. This metabolite inhibits the endothelial function and muscle vascularization, leading to ischemia, hypoxia, and finally the loss of muscle mass. Glutamine, a non-essential amino acid, is mainly catalyzed to ammonia and glutamate and is involved in many metabolic pathways. Patients with type 2 DM and sarcopenia can have impaired protein synthesis of glutamine, leading to reduced muscle protein synthesis of glutamine and resulting in decreased muscle protein synthesis, and thus making this metabolite a possible metabolic marker of diabetic sarcopenia. The isoxanthohumol levels were decreased in the patients type 2 DM with sarcopenia. However, isoxanthohumol administration can improve glucose and lipid metabolism [24] by reducing lipid accumulation which could decrease the risk of sarcopenia development in mouse model. Other metabolites that were found to be related with sarcopenia in type 2 DM were high 3-methylxanthine; high L-(+)-arginine, although a recent study reported that high levels might in fact enhance muscle protein synthesis [25]; and high D-gluconic acid, confirming previous research indicating gluconic acid as potential biomarker of sarcopenia [26].

In a prospective study conducted on a cohort of Japanese patients (*n* = 99) aged ≥65 years old diagnosed with type 2 DM, targeted metabolomic analysis established several amino acids as potential biomarkers of sarcopenia. Seventeen serum metabolites were quantified using gas chromatography mass spectrometry (GC-MS), and leucine and glutamic acid were outstandingly associated with the clinical indicators of sarcopenia. Lower leucine and higher glutamic acid have been noticed in patients with decreased muscle strength, and leucine was also found to be correlated with reduced muscle mass. These data were supported by a positive correlation between serum leucine concentration and indicators of body composition, such as skeletal muscle mass index (SMI) and grip strength. At the same time, leucine was found to be related with muscle mass, and leucine and glutamic acid were identified to be linked with low muscle strength. Low serum glutamate was associated with an increased risk of sarcopenia regardless of age and glycated hemoglobin A1c levels, but leucine did not show a significant independent association (OR = 1.09, *p* = 0.008). Further adjustments for serum creatinine and inhibitors of sodium-glucose co-transporter 2 (SGLT2i) indicated that lower glutamic acid remained associated with a higher risk for sarcopenia. Moreover, data for other amino acids showed lower alanine, aspartic acid, and phenylalanine, and higher serine in the sarcopenic risk group, but the differences did not reach statistical significance. Also, an age-related decrease in serum concentration of valine, leucine, serine, and glutamic acid was observed within the cohort, validating previously reported trends of amino acid reduction in aging-associated sarcopenia and type 2 DM [27]. Overall, metabolomic data confirm the value of leucine [28] and glutamic acid [29] as potential biomarkers for sarcopenia risk.

The relationship between plasma amino acids profiles and sarcopenia represented the main objective in a cross-sectional study involving 160 elderly Japanese participants, of whom 40% were diagnosed with DM. Handgrip strength, walking speed, and muscle mass were assessed, and 28 participants met the criteria for sarcopenia. A total of 18 plasma human proteinogenic amino acids were assessed using precolumn derivatization high-performance liquid chromatography/electrospray mass spectrometry (HPLC/ESI-MS) in all patients enrolled in the study. After performing multivariate analysis, elevated plasma proline concentration was the only variable independently associated with sarcopenia. In the same study, histidine and tryptophan levels were lower and glutamine concentration was higher in sarcopenic vs. non-sarcopenic persons. These findings could suggest that increased plasma proline reflects metabolic impairments linked to muscle degradation or serves as a marker for early sarcopenia detection. The pathophysiological explanation for these results is not fully elucidated, but some hypotheses suggest increased proteolysis or proline’s role in neuromuscular dysfunction and oxidative stress [30].

The FRAILMar study evaluated sarcopenia and frailty in patients with chronic kidney disease candidates for kidney transplantation (*n* = 173), of whom 38.2% were diagnosed with DM. Using unsupervised multi-modal integration, the study results indicated a high-risk metabolic phenotype composed of DM, sarcopenia, and low body mass index. Patients presenting with this phenotype exhibited alterations in serum levels of several biomarkers out of the total 75 biomarkers measured by liquid chromatography coupled with tandem mass spectrometry. Thus, significantly lower levels were found for three branched-chain amino acids (BCAAs): leucine, valine, and isoleucine, and for the aromatic amino acids: phenylalanine, tryptophan, tyrosine, and glutamate [31].

In the Health ABC study, Yao et al. investigated the metabolic mechanisms of unintentional weight loss in older adults aged 70–79 years (*n* = 1536), of whom 39% were diagnosed with DM, finding that metabolites associated with unintentional weight loss differed from those of intentional weight loss and weight gain. The authors measured 442 plasma metabolites using liquid chromatography mass spectrometry (LC-MS). Metabolites associated with unintentional weight loss were implicated in amino acid metabolism, mitochondrial dysfunction, and inflammation. BCAAs and aromatic amino acids were negatively associated with unintentional weight loss. Given the implication of BCAAs and aromatic amino acids in protein synthesis, energy production, and protein catabolism inhibition, the authors suggested the significant role played by muscle wasting in unintentional weight loss. In addition, the presence of DM could have explained many metabolites association and its role in unintentional weight loss should be considered [32].

Saoi et al. evaluated the adaptative metabolic response to changes in physical activity in older persons with prediabetes at risk of sarcopenia (*n* = 17) by non-targeted metabolomics using multisegment injection capillary electrophoresis mass spectrometry (MSI-CE-MS). The authors showed that after 2 weeks of step reduction followed by 2 weeks of recovery, there was an increase in several circulatory metabolites and a decrease in protein degradation and energy metabolism. One of the metabolites that increased after step reduction was glutamine, and it remained increased even in the post-recovery period. This was not the only metabolite which rose in this period, but also creatine and methionine were reported as being increased. In contrast, there were three different metabolites which had a decreasing trend during step reduction: indoxyl sulfate, hippuric acid, and oxoproline [33].

In a study conducted by Low et al., 1140 subjects with type 2 DM, with a mean age of 56.6 years, were followed for a mean period of 3.4 years to establish the changes in skeletal muscle mass, in close relationship with sarcopenia. Surprisingly, over 40% of the patients included in the study presented muscle loss during the follow-up period. Regarding the changes that were observed using targeted metabolomics with liquid chromatography tandem mass spectroscopy (LC-MS/MS), the authors reported high levels of three BCAAs, valine, leucine, and isoleucine, to be associated with higher SMI. Also, these amino acids were associated with decreased chances of muscle loss. These associations were still statistically significant after adjusting for age, ethnicity, sex, DM duration, and SMI at baseline. On the other hand, there were three different amino acids linked with greater muscles loss as higher values of glycine, arginine, and citrulline were seen in persons with muscle loss compared with those without muscle loss, while alanine and tryptophan were reported as being associated with muscle preservation. Interestingly, the evidence provided by this study regarding the significant positive association between BCAA and muscle preservation was present only in people younger than 60 years of age, while only proline was protective in older age [34]. The results highlighted by this study regarding the lower levels of isoleucine, leucine, and valine connected to muscles loss were confirmed in more recent studies [35,36,37], although even higher levels were also shown in previous studies [38,39].

In a type 2 DM-related sarcopenia mouse model, male diabetic mice (*db/db*) and nondiabetic mice (*db/m*) used as control mice were used for proteomics analysis to reveal insights of the role of lipid metabolism in type 2 DM-related sarcopenia. Gastrocnemius muscle mass, index, and strength parameters were remarkably smaller in severe obesity and diabetes *db/db* mice than in control *db/m* mice. Histological analysis confirmed the previous observations by revealing atrophic muscle fibers in *db/db* mice. Proteins were extracted from gastrocnemius muscle, and peptides obtained after trypsin digestion were analyzed using LC-MS/MS analysis and 4D mass spectroscopy. Out of 2029 proteins quantified, a total of 199 proteins were identified as differentially expressed proteins (DEPs) divided into 68 downregulated and 131 upregulated. The identified DEPs were involved in several metabolic pathways, particularly in lipid metabolism. Using gene set enrichment analysis to uncover potential disease mechanisms and to evaluate the functional enrichment of the proteins identified, the authors found that the proteins identified were remarkably enriched in “fatty acid oxidation”, “fatty acid catabolic process”, “lipid oxidation”, “peroxisome organization”, “establishment of protein localization to peroxisome”, and “protein targeting the peroxisome”. In addition, gene set enrichment analysis indicated that fatty acid oxidation-related pathways were upregulated in the model of type 2 DM-related sarcopenia. Also, it was found that the fatty-acid-oxidation pathway might play an important role in type 2 DM-related sarcopenia progression [40].

A prospective study for 8 weeks involving *db/db* mice (model for overeating and type 2 DM) and *db/m* mice conducted various analyses, including gene expression in skeletal muscle and metabolites in skeletal muscle and serum. Mice in the *db/db* group presented with lower muscle strength. The authors noticed that serum alanine, oxalic acid, malonic acid, succinic acid, and serine and citric acid measured using gas chromatography mass spectrometry were increased in *db/db* mice compared to *db/m* mice. These compounds are notable for their function in maintaining muscle mass and stimulating protein synthesis. In contrast, serum valine, isoleucine, glycine, leucine, threonine, aspartic acid, lysine, methionine, and phenylalanine were lower in *db/db* mice than in *db/m* mice [41].

The effect of aerobic exercise on gastrocnemius and soleus muscles was evaluated in exercise-trained *db/db* mice (*n* = 4) for 6 weeks compared with sedentary *db/db* mice (*n* = 4). Both muscles were prepared for proteomics analysis using LC-MS/MS at the end of the experiment and an increased cross-sectional area of the gastrocnemius muscle was found, while no significant changes were observed in the soleus muscle. Blood glucose and glycated hemoglobin A1c were significantly lower in the exercise group. The molecular mechanism involved in disease progression and DEPs were investigated using Ingenuity Pathway Analysis and functional enrichment analysis. Out of 3426 proteins found in gastrocnemius muscle, there were 155 DEPs in the exercise-trained group divided into 52 downregulated DEPs and 103 upregulated DEPs. Several proteins levels were found to be decreased in the gastrocnemius muscle and interpreted as an expression of the downregulating transforming growth factor-β signaling pathway, having muscle regeneration and remodeling as positive consequences [42].

Over 600 new as well as known metabolites from amino acid, peptide, and protein metabolism were reported in studies using untargeted and targeted metabolomic techniques. Interestingly, several known amino acids (valine, glycine, alanine, and proline) displayed a dual behavior (Table 1). Although metabolomics has a huge potential in identifying biomarkers and yields valuable information, the study populations as well as diseases severity might have influenced the presented results. Untargeted metabolomics uncovered multiple differentially expressed protein upregulated and downregulated in the presence of DM and sarcopenia.

### 2.2. Lipids

Lipids have been shown to play a significant role in sarcopenia, particularly in the context of myosteatosis and metabolic disturbances. Patients with DM frequently present lipid metabolism disturbances. The changes in the lipid metabolism found in the association of DM with sarcopenia were evaluated using metabolomic analysis to find how they can contribute to diseases development and progression.

Tan et al. found that patients with type 2 DM and sarcopenia presented significantly increased levels of serum pentadecanoic acid, a long-chain saturated fatty acid. Pentadecanoic acid is known for its ability to accumulate in the muscle, contributing to inflammation, lipotoxicity, and disordered protein synthesis, with all these mechanisms leading to muscle atrophy [43]. In addition, ectopic accumulation of long-chain saturated fatty acids can determine metabolic dysfunction and insulin resistance [44], which are well-known factors for sarcopenia and may represent a predictive biomarker for DM-related sarcopenia [22]. The association of sarcopenia with non-alcoholic fatty liver disease (NAFLD), a condition linked to long-chain saturated fatty acids, was investigated by serum metabolomic profile. The authors found that sarcopenic lean NAFLD patients were characterized by a different metabolome profile from the non-sarcopenic lean NAFLD and the overweight NAFLD, despite similar prevalence of DM. Thus, the investigation revealed upregulated low-density lipoproteins, triglycerides, valine, lactic acid, and reduced high-density lipoprotein 4, generating the hypothesis that this metabolomic profile might be incriminated for the higher risk of atherosclerotic carotid plaque and liver fibrosis in sarcopenic lean NAFLD patients [45].

Several sphingolipid metabolites, including sphingomyelins and ceramides, were associated with sarcopenia. In men with sarcopenia, sphingomyelins (16:1) and ceramide (24:1) were significantly higher than in men from the control group. In addition, sphingomyelin (16:1) exhibited the area under the receiver operating characteristic curve (AUROC) for low muscle strength and low muscle mass in men of 0.600 (95% CI: 0.501–0.699) and 0.647 (95% CI: 0.557–0.737), respectively. Meanwhile, sphingomyelins (24:1) and ceramide (24:1) exhibited the AUROC for low muscle strength and low muscle mass in men of 0.669 (95% CI: 0.581–0.757) and 0.670 (95% CI: 0.582–0.759). Conversely, no differences were observed between women with and without sarcopenia. Sphingolipid score, which resulted from an equation combining ceramide (24:1) and sphingomyelin (16:0), was found to have predictive ability for sarcopenia in the validation study cohort [46].

Gonzalez-Freire and colleagues evaluated in the Baltimore Longitudinal Study of Aging a total of 504 adults over 50 years of age, of whom 11% were diagnosed with DM. The participants were followed for 8 years and the authors determined plasma metabolites in order to correlate them with standardized walking speed, decline in walking speed, and gait assessed during numerous clinical visits. Targeted mass spectrometry analyzed 148 metabolites, of which, after adjusting for age and sex, 8 metabolites were associated with baseline gait speed. Three different lysophosphatidylcholines (17:0; 18:1; 18:2), phosphatidylcholine, and three different sphingomyelins (16:1; 18:0; 18:1) corresponded to lipid metabolism, but, after further adjustments for anemia and chronic conditions, lysophosphatidylcholine (18:2) was the only predictor for slower gain decline. These results emphasize that lysophosphatidylcholines (18:2), a lipid derived from linoleic acid, could be used as a biomarker for mobility deterioration associated with aging [47]. This metabolite was previously shown to be associated with other chronic diseases seen in older age as type 2 DM, demonstrating its value as a potential important biomarker for aging and age-related conditions [48,49].

A study including a natural aging mouse model of sarcopenia, cell models, and human participants older than 65 years in two cohorts searched for potential biomarkers of sarcopenia. The human discovery cohort, mostly diagnosed with DM (>90%), consisted of men (*n* = 72 cases and *n* = 72 controls) and women (*n* = 36 cases and *n* = 36 controls). The validation cohort consisted of men (*n* = 36 cases and *n* = 128 controls). Targeted and non-targeted metabolome profiling consisted of metabolites extraction using liquid–liquid extraction procedure and further determination using an LC-MS/MS system. In mice, a total of 146 metabolites out of 699 metabolic features were found to be significantly different between aged and young mice. Aged mice displayed higher plasma ceramide (C24:1) and muscle ceramide (C18:1), and metabolites inversely associated with muscle mass. Since sphingolipid metabolism was found to be potentially related to sarcopenia, it was selected for further analysis in muscle cells and human participants [46]. Significantly higher ceramides (14:0), (16:0), and (24:0) and sphingomyelin (24:0) in cell lysates were found in myocytes than in myoblasts; meanwhile, there was no difference between myotubes and myoblasts. Conversely, ceramide (20:0) and sphingomyelin (24:0) were significantly higher in myoblasts than in myotubes. Most ceramides and sphingomyelins decreased during cell differentiation [46].

Zhang et al. used male mice in order to address soleus muscle lipid profiles using shotgun lipidomics coupled with multidimensional mass spectrometry. The mice were either young, 6 months, or older mice, between 22 and 24 months. These mice were divided into three different groups: control, hind limb unloading for 10 days, or hind limb unloading for 10 days followed by 3 days of reloading recovery. The sarcopenic phenotype of the older mice was evidenced by the lower weight of the soleus muscle compared to younger mice. Also, old mice did not regain their insulin sensitivity, which was evaluated through low glucose clearance rate after an unloading and reloading period. However, there was no difference between mice in terms of age when it comes to soleus muscle loss after the intervention period. The study showed that in older mice unloading reduced fatty acid oxidation, demonstrated by a significantly decreased of long-chain acylcarnitines. Interestingly, the reloading period kept the long-chain acylcarnitines decreased. Also, during reloading there was an increasing trend seen for alfa-ketoglutarate, malate, and fumarate, suggesting that mitochondrial respiration uses glycolysis and anaplerosis [50]. Although aging and type 2 DM contribute to muscle loss, muscle atrophy due to disuse was similarly severe in both age groups, adult and old mice. However, the recovery was different depending on the group age as the older mice showed significantly impaired recovery. This was shown by mitochondrial dysfunction and increased levels of long-chain acylcarnitines [51,52]. These metabolic dysfunctions might affect protein synthesis by altering ATP (adenosine triphosphate) production and could affect anabolic response in the recovery period [53].

In a previous presented animal study, *db/db* mice (modeled for overeating and type 2 DM) showed higher serum and skeletal saturated fatty acids, myristic acid, palmitic acid, lauric acid, and stearic acid, compared to *db/m* mice. This muscle fatty acids accumulation was accompanied in contrast by a decreased level of those fatty acids in feces of *db/db* mice, a fact that can indicate an increased intestinal absorption. The level of palmitoleic acid and oleic acid, along with saturated fatty acids, were increased in serum and skeletal muscle in *db/db* mice but decreased in feces. This study also showed that the levels of acetic acid, butanoic acid, and propionic acid in the serum and feces of *db/db* mice were lower than those of *db/m* mice [41]. All mentioned saturated fatty acids are known to promote inflammation, a factor playing a pivotal role in sarcopenia pathology [21].

The investigation of lipid metabolism using untargeted and targeted metabolomic techniques identified most of the metabolites as increased in both humans and animal models with DM and sarcopenia compared to controls. Ceramides and sphingomyelins evaluated using untargeted metabolomics were found increased in humans and mice with DM and sarcopenia [46,47], and decreased in cell cultures [46] (Table 1). These results highlight the critical role played by lipid metabolism in mediating the relationship between DM and sarcopenia and further suggest their potential roles as biomarkers for disease progression as well as treatment targets.

### 2.3. Energy Metabolism and Carbohydrates

Although carbohydrates are not usually considered primary markers of sarcopenia, they have been associated with muscles health and sarcopenia development.

The previously introduced FRAILMar study evaluated sarcopenia and frailty in patients with chronic kidney disease candidates, of whom 38.2% were diagnosed with DM. Patients with DM, sarcopenia, and low body mass index presented with low pyruvate; meanwhile, lactase dehydrogenase was higher. Lactase dehydrogenase is an enzyme involved in anaerobic metabolism by interconverting lactate and pyruvate. Since pyruvate was lower, it means that lactate was higher. These imbalances indicate a complex impairment of amino acid metabolism, muscle, and energy metabolism, and it may reflect a remarkable disruption in the metabolomic profile for the subpopulation with chronic kidney disease candidates for kidney transplantation, where malnutrition and inflammation are prevalent [31].

The Baltimore Longitudinal Study of Aging, one of the biggest studies investigating plasma metabolites linked to muscle decline, included over 500 people (11% diagnosed with DM) and evaluated 148 plasma metabolites. Of the metabolites identified, hexoses, corresponding to carbohydrate metabolism were associated with baseline gait speed, but the significant association was not met after the follow-up period of 50.5 months and after multivariable adjusting for chronic diseases, anemia, age, and sex [47]. Also, in another study conducted on mice, malate, a TCA intermediate implicated also in gluconeogenesis, was reported as being increased during the reloading period [50]. In the Health ABC prospective study, the presence of DM attenuated by 30% the association between hexose and unintentional weight loss in older adults [32].

The skeletal muscle plays a significant role in the systemic metabolism. Kim et al. studied the role of Ubiquitin-specific protease21 (USP21) in skeletal muscle pathophysiology in a bioinformatic and experimental analysis. The function and mechanism of USP21 was evaluated using transcriptomics, lipidomics, and proteomics (LC-MS/MS USP21-interaction proteins) assays. The levels of USP21 in the skeletal muscle of a person with type 2 DM was higher than in a person without DM. Also, mice with a high-fat diet displayed in the skeletal muscle induction of USP21, and USP21 significantly correlated with blood glucose. In skeletal muscle-specific whole-body (KO) of USP21 the hyperglycemia induced by high-fat feeding for 9 weeks was significantly attenuated, while insulin sensitivity was improved. In skeletal muscle-specific USP21 knockout (MKO) and USP21-KO mice, muscle mass increased and energy expenditure was promoted through mitochondrial biogenesis and fatty acid oxidation. The authors concluded that USP21 was downregulated by exercise and upregulated by DM presence, and that USP21 might play a significant role in systemic energy homeostasis through influences on muscle mass, mitochondrial function, and heat generation [54].

The proteomic analysis of gastrocnemius muscle from diet-induced obesity animal model C56BL/J mice (*n* = 15 females, *n* = 15 males) fed with a standard or high-fat diet (45% or 60%) was performed using liquid chromatography coupled to electrospray tandem mass spectrometry (LC–ESI–MS/MS) and was followed by bioinformatic pathway analysis. Males exposed to high-fat diet during the 7 weeks experiment displayed higher glycemia and a significant increment in body weight. Protein extraction revealed the following significant differently changed proteins in males and in females: 160 out of 505, and 46 out of 580, respectively. Compared to the control group of males, 117 proteins were expressed differently in 45% high-fat diet and 131 in 60% high-fat diet. In females, 33 were expressed differently in 45% high-fat diet and 41 in 60% high-fat diet compared to the control group. Mitochondrial dysfunction was observed by identifying decreased respiratory chain complex I and V, decreased free fatty acids beta oxidation and tricarboxylic acid cycle (TCA), and increased complex III subunits which are involved in reactive oxygen species production, resulting in oxidative stress. Data resulting from glucose and glycogen metabolism and contractile proteins indicated increased switch from fiber-type slow/oxidative to fast/glycolytic. Changes in structural proteins indicated increased muscular stiffness [55]. The gender-specific approach in obesity and comorbidities such as type 2 DM could be considered given the fewer alterations in the activating mechanism meant to counteract the increase in fatty acids secondary to high-fat diet observed in female mice [55].

A prospective study that involved *db/db* mice (model for overeating and type 2 DM) and *db/m* mice that conducted various analyses noticed increased levels of intermediate metabolites of the Krebs cycle and glycogen metabolism that were recorded in serum of *db/db* mice, which suggests a partial blockage in oxidative energy metabolism and maybe a reaction to lipid accumulation and mitochondrial dysfunction [41].

The complexity of carbohydrate and energy metabolism was explored in a few targeted and untargeted metabolomic studies, revealing a clear difference between the participants with DM and sarcopenia and controls (Table 1). Untargeted metabolomics uncovered two enhanced metabolic pathways and two elevated intermediate metabolites in DM-related sarcopenia.

Table 1 synthetizes the serum metabolites from protein, lipid, carbohydrate, and energy metabolisms in sarcopenia and DM.

**Table 1:** Serum metabolites in sarcopenia and diabetes mellitus.

Metabolism	Serum Metabolites in Sarcopenia and Diabetes Mellitus	
	Increased Levels	Decreased Levels
Proteins	Targeted metabolomics: Branched-chain amino acids: valine [45] Other: alanine [41,56] arginine [34,56] L-(+)-arginine [22] citrulline [34] glutamine [22,30,33] glycine [34] proline [30] serine [27,41] D-gluconic acid [22] glutamic acid [27,56] 3-methylxanthine [22] 5′-methylthioadenosine [22] asymmetric dimethylarginine [22] N,N-dimethylarginine [22] Ubiquitin-specific protease21 [54] citric acid [41] oxalic acid [41] malonic acid [41] succinic acid [41] Untargeted metabolomics: 82 metabolites (overall) [22] 75 metabolites (overall) [31] 131 differentially expressed protein upregulated [40] 103 differentially expressed protein upregulated [42] 160 proteins (males), 46 proteins (females) [55]	Targeted metabolomics: Branched-chain amino acids: leucine [27,31,32,34,41] valine [31,32,34,41] isoleucine [31,32,34,41] Aromatic amino acids: phenylalanine [27,31,32,41] tryptophan [30,31,32,34] tyrosine [31,32] Other: alanine [27] glycine [41] glutamate [41] histidine [30] lysine [41] methionine [41] proline [34] threonine [41] isoxanthohumol [22] aspartic acid [27,41] Untargeted metabolomics: 68 differentially expressed protein downregulated [40] 52 differentially expressed protein downregulated [42]
Lipids	Targeted metabolomics: low-density lipoprotein triglycerides [45] Monounsaturated fatty acids: palmitoleic acid [41] oleic acid [41] Aaturated fatty acids: pentadecanoic acid [43] myristic acid [41] palmitic acid [41] lauric acid [41] stearic acid [41] Untargeted metabolomics: 146 metabolites (overall) [46] Ceramides (14:0), (16:0), (24:0) [46] sphingomyelin (24:0) [46] sphingomyelins (16:1), (24:1) [46] ceramide (24:1) [46] lysophosphatidylcholines (17:0; 18:1; 18:2) [47] phosphatidylcholine [47] sphingomyelins (16:1;18:0;18:1) [47]	Targeted metabolomics: - Untargeted metabolomics: most ceramides, sphingomyelins evaluated [46] long-chain acylcarnitines [50]
Carbohydrates and Energy	Targeted metabolomics: lactic acid [45] lactase dehydrogenase [31] respiratory chain complex III subunits [55] Untargeted metabolomics: fatty acid oxidation related pathways [40] intermediate metabolites of Krebs cycle [41] intermediate metabolites of glycogen metabolism [41]	Targeted metabolomics: Hexoses [32,47] alfa-ketoglutarate [50] malate [50] fumarate [50] pyruvate [31] respiratory chain complex I and V [55] free fatty acids beta-oxidation [55] tricarboxylic acid cycle [55] Untargeted metabolomics: -

### 2.4. Therapeutic Approaches

Compounds and medications known for their therapeutic benefits in DM, newly discovered metabolites using metabolomics analysis, physical exercise, targeted genetic interventions, as well as different surgical approaches, were all explored for their therapeutic potential using targeted and untargeted metabolomic techniques in humans and animals with DM and sarcopenia.

D-Pinitol, a compound found in plant-based diets, exhibited antidiabetic and insulin regulator properties in several preclinical and clinical studies [57,58]. Yu et al. investigated the mechanism of D-Pinitol in treating DM-related sarcopenia by high-throughput analysis of the 16S rRNA gene, metabolome, and proteome. D-Pinitol was administrated intragastrically to Streptozotocin-induced diabetes mice SAMP8 at a dose of 150 mg/kg for a total of 8 weeks. The muscle atrophy identified in diabetic mice was significantly alleviated when using D-Pinitol. A total of 261 metabolites and 626 proteins were significantly modified in the gastrocnemius muscle of diabetic mice evaluated using metabolome and proteome analysis. Among these, therapy with D-Pinitol restored to normal levels 17 proteins and 44 metabolites. The functional signaling pathways of diabetic mice treated with D-Pinitol included histidine and β-alanine metabolism, nucleotide metabolism, calcium signaling pathway, and ATP-binding cassette. In addition, fecal 16S rRNA gene sequencing identified dysfunction of gut microbiota in mice with diabetic sarcopenia. These data offer new insights and perspectives in the treatment of DM-related sarcopenia [59].

Following the identification of relevant metabolites using targeted and untargeted metabolomic analysis, their potential therapeutic use in clinical practice was evaluated in human and animals with DM and sarcopenia. Among them, ceramides were explored for their potential therapeutic success using metabolomics. Given the implications of sphingolipid metabolism in sarcopenia in both mice and human participants, of whom most were diagnosed with DM, muscles cells exposure to ceramides (16:0), (18:0), and (24:0) were assessed. It was found that this treatment decreased the viability of muscle cells and significantly decreased the fusion index and myotubes [46]. In line with this finding, several other studies described the negative regulator role of ceramides in myogenic differentiation [60,61]. Adjacent to their indicative role in sarcopenia, ceramides could also serve as treatment targets.

The implications of physical activity, a lifestyle factor, have been investigated in several metabolomic studies involving both humans and animal models of DM and sarcopenia, given the known positive effects on muscle mass and function. Aerobic exercise induced upregulated metabolic pathways through exercise-regulated DEPs in gastrocnemius muscle in a mouse model of type 2 DM-related sarcopenia, directly indicated by increased lipid and glucose metabolism, muscle regeneration, and insulin sensitivity. The authors concluded that engaging in 6 weeks of aerobic exercise might have a favorable effect on the recovery of type 2 DM-related sarcopenia [42].

In a study that evaluated patients with prediabetes and overweight at risk of sarcopenia (*n* = 17), non-targeted metabolomics showed fasting plasma metabolite changes after the intervention, which consisted of two weeks of reducing steps lower than 1000 steps/day. An elicited increase was observed in glutamine, carnitine, creatine, methionine, and deoxicarnitine during step reduction and remained persistently elevated even after recovery period. Meanwhile, oxoproline, indoxyl sulfate, and hippuric acid decreased during step reduction. To avoid any food interference with the metabolites, subjects received standardized meals for the last three days of the intervention. These findings suggest that longer periods of inactivity increased protein catabolism and reduced carnitine uptake of carnitine in the muscle, and also deoxycarnitine, which is a precursor of carnitine [33]. In the light of these findings, the authors questioned whether people with DM at risk for sarcopenia could be treated with dietary supplements, for example, glutamine, carnitine [62], and creatine [63], usually known to be dedicated for improving muscle health. But their efficacy in reversing or stopping muscle atrophy needs to be demonstrated. These findings raise the question of multiple interventions, such as resistance training coupled with protein supplementation, in order to reduce sarcopenia and promote healthy aging.

People suffering from sarcopenia present with abnormal muscle wasting as a consequence of increased protein breakdown in association with a metabolic disorder such as DM [64,65]. USP21 is highly expressed in skeletal muscle and plays a significant role in fuel consumption and oxidative capacity. After identifying USP21 ablations effect on mitochondrial fuel consumption and thermogenesis, Kim et al. investigated the effects of USP21-KO (ablation of USP21 in skeletal muscle) on obesity and type 2 DM in a mouse model compared with a control. Although fed with a high-fat diet, the KO mice resisted to weight gain induction as well as fat mass accumulation. The USP21-KO mice had a higher oxygen consumption and energy expenditure, despite having similar locomotor activities with the controls. USP21 ablation was proven to prevent obesity through increasing energy expenditure [54]. Thus, an important risk for type 2 DM represented by obesity might be modulated by genetic USP21 ablation in skeletal muscle.

The diabetes therapeutic landscape has been dominated in past years by glucagon-like peptide-1 receptor agonists (GLP-1 RA) and SGLT2i, medications that have demonstrated multiple metabolic, cardiovascular, renal, and possible neurological benefits [66,67,68]. One of the less studied favorable effects of the SGLT2i class is the reduction in fatty acid accumulation at the extracellular and intramuscular levels, while data from the literature pointed that increased concentration of intramuscular fatty acids has been associated with both impaired glucose metabolism and with a high risk of sarcopenia and sarcopenic obesity [69]. In this context, Bamba et al. assessed the effect of luseogliflozin, an SGLT2i, on skeletal muscle, serum lipidomes, as well as on skeletal muscle transcriptomes, in non-diabetic heterozygous male *db/m* mice and diabetic homozygous male *db/db* mice. The results demonstrated that in the group treated with SGLT2i there was a reduction in visceral adiposity accumulation (*p* = 0.004), and an increase in soleus muscle weight (*p* = 0.010) and grip strength (*p* = 0.0001). More than that, a decrease in palmitic acid concentration (a saturated fatty acid) and an increase in unsaturated fatty acid concentration, especially oleic acid, both in muscle and sera was observed as a result of SGLT2i administration. In addition to these modified fatty acid levels in skeletal muscle cells, the reduced expression of genes, such as Fasn, Elovl6, and Scd1 which are involved in fatty acid synthesis, was found. So, in this research, SGLT2i administration demonstrated beneficial effects in reducing obesity-dependent sarcopenia via modifying extracellular lipidome, which influenced intramuscular fatty acid metabolism and gene expression [70].

The concern that SGLT2i could induce muscle atrophy or sarcopenia associated with their weight loss effect is a topic of great interest. Thus, another team of researchers analyzed the effect of canagliflozin on muscle mass and function using nondiabetic mice. The results of this study demonstrated a differential effect of SGLT2i on slow and fast muscles, which was further influenced by the amount of food intake. It was observed that in the increased food intake condition SGLT2i induced increased fast muscle function, whereas slow muscle function was unaffected, although slow and fast muscle mass were maintained. Grip strength, which evaluates fast muscle function, was increased in canagliflozin-treated mice, but no significant difference was observed between the groups. During SGLT2 inhibition, amino acids and free fatty acids were increased in slow muscle but were unchanged in fast muscle. Moreover, glycolytic metabolites and ATP were increased in fast muscle, whereas glycolytic metabolites were reduced but ATP was maintained in slow muscle. The findings of this study may offer useful understanding of the appropriate use of SGLT2is in managing DM in patients at high risk of sarcopenia [71].

Another study explored the effects of SGLT2i on skeletal muscles in genetically diabetic *db/db* mice treated with canagliflozin for 4 weeks by measuring the running distance and handgrip strength. A targeted metabolome analysis of the skeletal muscles was also performed, using the *db/db* mice with and without canagliflozin treatment and *db/+* mice as reference. Canagliflozin significantly improved the running distance in *db/db* mice, revealing an enhancement in the previously reduced endurance capacity. In the soleus muscle, canagliflozin decreased glucose and increased acetyl-CoA and fatty acids; also, canagliflozin increased metabolites of the TCA (citrate, aconitate, isocitrate, fumarate, and succinate) and intermediates of glycolysis (glucose-6-phosphate and fructose-6-phosphate). In the extensor digitorum longus muscle, canagliflozin decreased glucose levels and slightly influenced glycolysis and TCA metabolites. In addition, canagliflozin upregulated several metabolites by more than two-fold in the soleus muscle of *db/db* mice; among them, 5-aminoimidazole-4-carboxamide-1-β-D-ribofuranosyl 5′-monophosphate (AICARP) was thought to be the mechanistic link between the improved endurance capacity and the observed changes in fatty acid oxidation. Thus, canagliflozin reversed the pathological changes in the dysregulated skeletal muscles of genetically diabetic *db/db* mice [72].

GLP-1 RAs demonstrated significant and beneficial weight loss in patients with type 2 DM and obesity. However, there is controversial data suggesting that GLP-1 RA might also accelerate muscle loss and prone to sarcopenia risk [73], although the percentage of lean mass relative to total body weight was reported to be unaffected [74]. Two weeks treatment with liraglutide, a GLP-1 RA, decreased fatty acids and partially restored carbohydrate and amino acid metabolism in the gastrocnemius muscle of mice fed with a high-fat diet and evaluated using LC-MS/MS [75]. Semaglutide reduced gastrocnemius intramuscular fat accumulation, increased protein synthesis, and increased the proportion and the function of skeletal muscle in obese mice treated for 12 weeks and evaluated using untargeted metabolomics [76]. Both GLP-1 RAs liraglutide and semaglutide improved skeletal muscle atrophy, reduced excessive lipid accumulation, and restored glucose intolerance in obese mice [77]. In humans with type 2 DM and obesity, three months treatment with liraglutide positively influenced the plasma glucoronate interconversion pathway and several amino acids metabolism [78]. Further data from studies evaluating the effects of GLP-1 RAs in patients with type 2 DM and sarcopenia using metabolomics are needed to confirm their beneficial effects. Given the findings regarding SGLT2i and GLP-1 Ras in DM and sarcopenia, their use in clinical practice might be considered in addition to the already known cardiorenal benefits currently recognized in clinical guidelines.

The interconnection between pre- and postoperative muscle loss and different major surgical interventions has been a concern and a topic of interest for many researchers. In this retrospective study performed on 64 matched pairs, of whom 20–25% were diagnosed with DM, the authors compared the extent of muscle loss between conventional open liver resection and laparoscopic liver resection. The results showed that open liver resection was associated with significantly greater reduction in the psoas muscle index and a higher incidence of significant postoperative muscle loss. Open liver resection and type 2 DM were independent risk factors in multivariate analysis for significant muscle loss. Metabolomic profiling using LC-MS analysis revealed reduced serum citrulline and ornithine levels in patients with substantial muscle loss, suggesting altered urea cycle function. The findings support laparoscopic liver resection as a preferable surgical approach, with potential benefits in preserving muscle mass, reducing early recurrence, and maintaining metabolic integrity [79].

The studies exploring the potential benefits of several therapies and interventions using metabolomics might improve the clinical approach of DM-related sarcopenia (Table 2). Thus, treatment with GLP-1 RA and SGLT2i could be considered in addition to already known benefits in patients with DM. Compounds, such as D-pinitol, could be evaluated in future studies to assess adequate dosage and safety in humans. USP21 ablation using gene knockout ablation proved to be effective in increasing energy expenditure in skeletal muscle in DM. Conversely, metabolites, such as ceramides, could be used as treatment targets given their negative role in DM-related sarcopenia. Physical activity, an important lifestyle factor, is already recognized for health benefits, and the presented studies confirmed its positive influence on DM-related sarcopenia [33,42]. As expected, invasive surgical intervention aggravated the prognostics in DM-related sarcopenia, but it could be further explored given the evidence is limited to one study. Although the role of medical nutrition therapy of sarcopenia in DM has been previously described [80], only the studies using metabolomic analysis techniques were included in this review.

**Table 2:** The effect of therapies and interventions in diabetes mellitus associated with sarcopenia.

Therapy/Intervention	Effect on Diabetes Mellitus and Sarcopenia
D-pinitol	favorable [59]
Ceramides	unfavorable [46]
Aerobic exercise	favorable [42]
2 weeks step reduction, followed by 2 weeks recovery	unfavorable [33]
Genetic USP21 ablation	favorable [54]
Inhibitors of sodium-glucose co-transporter 2 (luseogliflozin, canagliflozin)	favorable [70,71,72]
Glucagon-like peptide-1 receptor agonists (liraglutide, semaglutide)	uncertain, probably positive [73,74,75,76,77,78]
Conventional open surgical intervention vs. laparoscopic intervention	unfavorable [79]

This narrative review has several limitations. First, we selected the studies based on several keywords and we did not find enough literature to perform a systematic review. We did not assess the quality, design, or limitations of the selected studies. We did not take into account the bias related to the sex, race, and age in studies involving human participants. Given the low number of studies and the high variability of data published in studies involving humans, animal models, and cell cultures, we described the current state of knowledge in this field. Heterogeneous results might have been related to the biological samples analyzed in the described studies (plasma, serum, skeletal muscle, and feces). Challenges are also raised by interpreting data coming from different species evaluated using multiple metabolomic techniques, with few studies disclosing analysis specificity and sensitivity. Future studies might bring enough evidence to perform a more precise synthesis.

## 3. Conclusions and Further Needs

Recent evidence from targeted and untargeted metabolomic studies showed that sarcopenia in DM is associated with significant changes in protein, lipid, carbohydrate, and energy metabolisms. Several metabolomic markers, as well as promising interventions for slowing the progression of sarcopenia associated with DM, have been identified in the literature. Newer biomarkers were reported using untargeted metabolomics. Known metabolites were also found significantly modified, while several known amino acids and lipids identified using targeted and untargeted metabolomics, respectively, displayed a dual behavior. Both their role as biomarker and therapy target of ceramides could warrant further exploration. The use of SGLT2i and GLP-1 RAs could be considered in the light of the presented studies, in addition to their proven cardiorenal benefits. Among lifestyle factors, physical activity had a positive influence and could be encouraged for slowing the progression of sarcopenia in DM. However, there are several limitations of the current evidence, such as the cross-sectional design of many studies, differences in operational definitions of sarcopenia, different types of DM, and the lack of the full validation of metabolites identified using untargeted metabolomics. Further research is needed to confirm the utility of these findings and to provide potential metabolomic biomarkers that might be relevant for the pathogenesis and treatment of sarcopenia associated with DM.

## Data Availability

No new data were created or analyzed in this study.

## Abbreviations

The following abbreviations are used in this manuscript:

AUROC	Area under the receiver operating characteristic curve
ATP	Adenosine triphosphate
BCAA	Branched-chain amino acids
CI	Confidence interval
DALYs	Disability-adjusted life years
DEP	Differentially expressed protein
DM	Diabetes mellitus
GC-MS	Gas chromatography mass spectrometry
GLP-1 RA	Glucagon like peptide-1 receptor agonists
HPLC/ESI-MS	Precolumn derivatization high-performance liquid chromatography/electrospray mass spectrometry
LC–ESI–MS/MS	Liquid chromatography coupled to electrospray tandem mass spectrometry
LC-MS	Liquid chromatography mass spectroscopy
LC-MS/MS	Liquid chromatography tandem mass spectroscopy
MSI-CE-MS	Multisegment injection capillary electrophoresis mass spectrometry
NAFLD	Non-alcoholic fatty liver disease
OR	Odds ratio
ROC	Receiver operating characteristic
SGLT2i	Inhibitor of sodium-glucose co-transporter 2
SMI	Skeletal muscle mass index
TCA	Tricarboxylic acid cycle
UHPLC-ESI-MS/MS	Ultra-high-performance liquid chromatography electrospray-ionization tandem mass spectroscopy
UPLC/MS	Ultraperformance liquid chromatography/mass spectrometry
USP21	Ubiquitin-specific protease21
